# Supplementary material for: Comparison of thoracic ultrasonography (TUS), clinical respiratory scoring (CRS), and blood analysis to evaluate respiratory dysfunction in transported calves
Source: PLoS One. 2026 Feb 11;21(2):e0337418. doi: 10.1371/journal.pone.0337418 (PMC12893609; doi:10.1371/journal.pone.0337418)
Supplement: Attachment — Submitted filename: Review PLOS.docx [file pone.0337418.s002.docx]

TUS and CRS comparisons with blood are made despite blood being sampled on different days than clinical assessments (T2, T4, T5). This can lead to inconsistencies and should be addressed with caution or sensitivity analysis.

Clarify this limitation in the methods more explicitly and consider whether statistical correction or subgroup analysis might mitigate this mismatch.

Some values were excluded due to suspected lab errors (e.g., IgM, eosinophils). However, no mention is made of imputation or sensitivity analyses.

The kappa value (0.038) indicates **no meaningful agreement**, yet this is only discussed briefly. This result is central to the paper's conclusions

**Grouping TUS score 4 into score 3**:

- This collapsing may affect results significantly given the small number of TUS 4 observations. A sensitivity analysis keeping TUS 4 separate, or pooling with discussion of clinical relevance, would improve rigor.

Use more concise language in the abstract and results.

“...followed by a gradually decrease” → should be “a gradual decrease”

“...severe signs of respiratory disease” → could be simplified to “severe respiratory signs”

"The CRS system was based on the Wisconsin calf health scorer..." → should be “based on the Wisconsin Calf Health Scoring Chart”

Ensure past tense is used consistently in the methodology (e.g., "calves were scored" vs. "calves are scored")

Several sections (especially Discussion) contain overly long paragraphs. Breaking these into thematic blocks would improve clarity.

This is a scientifically sound manuscript with good potential. However, I recommend a **major revision** focusing on:

- Statistical robustness and discussion of multiple comparisons.
- Clearer language and grammar corrections.
- Enhanced explanation of mismatched sample timing and potential biases.
- A clearer discussion of the CRS vs. TUS discordance and implications for field diagnosis.
