# Supplementary material for: Comparison of thoracic ultrasonography (TUS), clinical respiratory scoring (CRS), and blood analysis to evaluate respiratory dysfunction in transported calves
Source: PLoS One. 2026 Feb 11;21(2):e0337418. doi: 10.1371/journal.pone.0337418 (PMC12893609; doi:10.1371/journal.pone.0337418)
Supplement: Attachment — Submitted filename: Correction.docx [file pone.0337418.s003.docx]

**Comparison of thoracic 1 ultrasonography (TUS), clinical 2 respiratory scoring (CRS), and 3 blood analysis to evaluate 4 respiratory dysfunction in 5 transported calves**

1 . L31 | "TUS scores (0: healthy to 3: severe consolidation)" – Incomplete scale; Table 2 shows scores up to 5. | Revise to: "TUS scores (0: healthy to 5: severe pneumonia)" and clarify in text that scores 4–5 were rare and collapsed into score 3 for analysis (L196–197). |

2 . L32 | "CRS (0: healthy to 3: severe clinical symptoms)" – Implies linear scale, but scoring is categorical. | Clarify: "CRS categories (0: healthy, 1: mild, 2: moderate, 3: severe)" to reflect ordinal nature.

3 . L35 "3 or 7 days post-arrival (T3)" – Introduces variability without justification. | State: "T3 was defined as 3 or 7 days post-arrival due to logistical constraints; analysis adjusted for time."

4 . L41 "Total immunoglobulins and IgG were lower for TUS of 3" – IgG is part of total Ig; cannot be lower unless others decrease. | Recheck calculation: Total Ig = IgA + IgG + IgM. If IgG ↓ and total Ig ↓, clarify if IgA/IgM compensate.

5 . L59 "Calves that are diseased pre-transport should not travel" – Assumes causality not tested. Temper: "Calves with clinical signs pre-transport may be at higher risk during transport."

6 . L65 "CRS has low sensitivity (0.27–0.64)" – No citation for these values in transport context. | Cite source specifically for transport or pre-transport settings (e.g., Donlon et al. 2023, Ref 21).

7 . L74 "TUS has sensitivities >0.85 and up to 0.94" – Cites [10,11], but [10] is subclinical, [11] lacks necropsy validation. Specify: "Sensitivities vary by population; in subclinical calves, Ollivett et al. [10] reported 0.85–0.94 vs. BAL, not necropsy."

8 . L89–94 Calves from farms and marts mixed without accounting for origin differences. Include "origin (farm vs. mart)" as a covariate in models or stratify analysis.

9 . L95–98 Describes cohort differences (sex, age, breed) but no statistical adjustment. Adjust models for cohort (C1 vs C2) or include as random effect.

10 . L105 "Calves from livestock mart assessed on day of transport" – Timing differs from farm calves (L104: one day pre). Standardize or acknowledge bias in pre-transport assessment timing.

11 . L122–124 All calves received antibiotics upon arrival – confounds disease progression. Acknowledge that metaphylaxis limits natural disease progression and affects TUS/CRS/blood interpretation.

12 . L130–133 TUS and CRS not performed on same day at T4/T5 (blood sampled 1–2 days apart). State: "Non-simultaneous sampling may reduce correlation; results interpreted with caution."

13 . L134–136 No blood at T3, but TUS/CRS done – limits temporal comparison. | Justify gap and avoid claiming integrated assessment at T3.

14 . L147 "Depth of 11cm" – Not validated for calf thorax; may miss deep lesions. Cite justification or note potential for missed consolidations.

15 . L153 "6-tier TUS" – Inconsistent with Table 2 (6-point scale) and text (0–3 used). Correct: "A 6-point TUS scale was used, but scores 4–5 were combined with 3 due to low frequency."

16 . L161–163 | Rectal temperature omitted at T1 and imputed at T4 – introduces bias. Exclude temperature from T1 CRS or use alternative scoring; do not impute without validation.

17 . L167–168 CRS categories based on sum 0–1=0, 2–3=1, etc. – unequal binning. Justify binning or cite Wisconsin system for this grouping.

18 . L177 "26ml blood" – excessive volume for young calves (~28d). Confirm volume is safe (<10% total blood volume); typical calf blood volume ~80 mL/kg. 26 mL from 55kg calf = ~0.5% – acceptable, but state safety.

19 . L182–183 Two hematology analyzers used (Advia 2120, XT-1800i) – may introduce batch effects. Perform cross-calibration or include analyzer as covariate in models.

20 . L187 Hb units converted (g/dL to mmol/L) – conversion factor not cited. Cite: "Hb converted using factor 0.6205 (mmol/L per g/dL) [Ref]."

21 . L195–196 One calf removed due to missing TUS – violates intention-to-treat. Report as missing data and use imputation or sensitivity analysis.

22 . L196–197 TUS 4 reclassified as 3 – alters disease severity. Report both ways: primary analysis with TUS 0–3, secondary with TUS 4 separate.

23 . L198–200 Outliers removed without statistical justification (e.g., >3 SD). Define outlier criteria (e.g., >3 IQR) and report number excluded.

24 . L207–212 "Minimal model" not defined; no random effects for repeated measures. Use mixed models with calf ID as random effect to account for repeated measures.

25 . L212 Residuals tested for normality – but no mention of transformation validation. Show Q-Q plots or Shapiro-Wilk results in supplement.

26 . L214–220 Different transformations per variable – but no consistency in back-transformation. Ensure all SEs and CIs are correctly back-transformed (L224–229 are correct; verify in tables).

27 . L222 Weighted kappa used – appropriate, but no confidence interval reported. Add 95% CI for kappa in text or figure.

28 . L241 "No calves died" – but mortality is relevant to BRD severity. Discuss survival bias: severe cases may have been treated early.

29 . L243 "67% had TUS ≥2" – implies disease, but no validation against gold standard. Use: "67% had ultrasound findings suggestive of lung pathology."

30 . L244–248 Location of lesions described, but not analyzed statistically. Report if right cranial lobe predominance is significant (e.g., chi-square).

31 . L257 "Chi-square; p=0.03" – Chi-square for transition frequencies? Inappropriate. Use McNemar’s test or generalized estimating equations (GEE) for paired ordinal data.

32 . L289 "Chi-square; p<0.01" – same error as L257. Replace with GEE or marginal model for longitudinal CRS changes.

33 . L307 "9 out of 14 variables changed" – cherry-picking; no adjustment for multiple testing. Apply FDR correction (e.g., Benjamini-Hochberg) and report q-values.

34 . L308 "Neutrophil/lymphocyte ratio decreased between T4 and T5" – contradicts Table 4 (T3 missing). Correct: "N/L ratio decreased from T1 to T5" or clarify trend.

35 . L315–316 Table 4: HGB different superscripts (a,b) but p= <0.01 – correct, but T5 lower than T2/T4? Verify post-hoc test: T5 is lowest, should be 'c' if significant.

36 . L322 "Haemoglobin did not show differences despite significant p=0.04" – contradiction. Recheck: p=0.04 is significant; either remove "did not show" or correct p-value.

37 . L323 "Lymphocyte count was lower for TUS of 3 than any lower TUS" – but TUS 0 vs 1 not different? Specify: "significantly lower than TUS 0, 1, and 2".

38 . L324 "N/L ratio higher for TUS 3 vs 0 or 2" – but p=0.03 and 0.01 – significant, but not vs 1? Clarify: "not significantly different from TUS 1".

39 . L325 "SAA higher for TUS 2 or 3 than TUS 1" – but Table 5 shows TUS 1=49.9, TUS 2=65.4, TUS 3=73.3. Correct: "higher for TUS ≥2 than TUS 0 or 1".

40 . L326 "Total Ig lower for TUS 3 than TUS 2" – but TUS 1=14.2, TUS 2=16.4 – TUS 1 not different? State: "significantly lower than TUS 2", not "any lower TUS".

41 . L331 "Haemoglobin did not show differences" – but p=0.04 – error. Correct: "Haemoglobin decreased with increasing TUS (p=0.04), though post-hoc differences were not significant."

42 . L341 "Haemoglobin lower for CRS 1 than 0" – but Table 6 shows CRS 2 and 3 also lower. Say: "lowest at CRS 1 and 2", or recheck pairwise tests.

43 . L344 "Eosinophil count did not show differences despite p=0.05" – p=0.05 is significant. Correct: "marginally significant" or adjust alpha.

44 . L355 "Severe TUS never correlated to severe CRS" – but n may be too small. Add: "though low frequency of severe cases limits inference."

45. L377 "almost 50% had some inflammation" – but TUS 1 may not be pathological. Use: "ultrasound abnormalities", not "inflammation", without histology.

46 . L380 "prevalence of severe lesions 4.8%" – based on observations, not calves. Report per calf: "4.8% of calves had at least one TUS=3 or 4 observation."

47 . L382 "72% of study calves displayed lung lesions" – at what time? T3? Specify: "at T3, 72% of calves had TUS ≥1."

48 . L388 "consolidation to fully recover" – assumes reversibility without histology. Say: "ultrasound lesions resolved", not "fully recover".

49 . L394 "failure of ultrasound technique" – contradicts claim of high sensitivity. Replace: "inter-observer variability or transient lesions may explain changes."

50 . L400 "rectal temperatures omitted... due to practical concerns" – weakens CRS validity. Acknowledge CRS is incomplete pre-transport and may underestimate disease.

51 . L416–418 "174 observations of healthy CRS, 104 not healthy by TUS" – but TUS may detect subclinical disease. Reframe: "TUS detected abnormalities in 60% of clinically healthy calves."

52 . L430 "haemoglobin usually increases" – incorrect; Hb often decreases in BRD due to anemia of inflammation. Correct: "In contrast to some reports, haemoglobin decreased, possibly due to acute phase response."

53 . L439 "Low total Ig and IgG... more at risk" – cites [16], but [16] is about arrival Ig predicting disease. Accurate: "As shown by Pardon et al. [16], low Ig at arrival predicts later BRD."

54 . L463 "TUS requires up to five minutes" – but no data in manuscript supports this. Add: "Based on observer logs, average scan time was X minutes."

55 . L465 "scanning right cranial lobe captures 61% of severe scores" – useful, but not validated. Recommend: "may be sufficient for screening, pending validation."

56 . L476–478 Blood and TUS/CRS not simultaneous – major limitation for correlation. State: "non-synchronous sampling likely attenuated correlations between blood and clinical scores."

57 . L481–482 "suboptimal situations" – but no description of how this affected blinding or consistency. Describe environmental constraints and their potential impact on scoring reliability.

58 . L484 "observers wear respiratory masks" – safety note, not research finding. Move to methods or supplementary.

59 . L485 "study increased stress" – confounder not measured or adjusted for. Measure cortisol or behavior, or acknowledge as limitation.

60 . L487–497 Conclusion: "TUS should be compared to other methods" – already done. Reframe: "TUS showed better alignment with immune markers than CRS, supporting its use in high-risk settings."
