# Supplementary material for: Comparison of thoracic ultrasonography (TUS), clinical respiratory scoring (CRS), and blood analysis to evaluate respiratory dysfunction in transported calves
Source: PLoS One. 2026 Feb 11;21(2):e0337418. doi: 10.1371/journal.pone.0337418 (PMC12893609; doi:10.1371/journal.pone.0337418)
Supplement: Attachment — Submitted filename: Reviewer responses_2025.10.13.docx [file pone.0337418.s004.docx]

**Dear Dr. Mahmoud,**

Thank you for taking the time to consider our manuscript. We have carefully reviewed the comments provided by the reviewers and have revised the manuscript accordingly. Detailed, point-by-point responses to all reviewer comments are included.

In addition, we have addressed a few additional queries related to the journal’s submission requirements, as outlined below.

1) Please ensure that your manuscript meets PLOS ONE's style requirements, including those for file naming.

- We have amended the file naming and reviewed the journal requirements once more, we hope the manuscript now conforms to the requirements

2) To comply with PLOS ONE submissions requirements, in your Methods section, please provide additional information regarding the experiments involving animals and ensure you have included details on (a) methods of sacrifice, (b) methods of anesthesia and/or analgesia, and (c) efforts to alleviate suffering.

- As this was an observational study, we were not in charge of providing euthanasia or analgesia to study animals. They were housed in a commercial environment. We have included the following statement in the methods to clarify this issue: “No animals were euthanized for this study. Analgesia or euthanasia was provided only at the discretion of the farmer and attending veterinarian, and all handling was performed to minimize stress.”

3) We noted in your submission details that a portion of your manuscript may have been presented or published elsewhere. “The blood data presented in this paper has previously been reported in two separate papers (attached as Paper 1 and Paper 2), and the CRS data has been reported in Paper 2 in a different format. However, the current paper is the first to incorporate TUS data, and it is the only one in which multiple methods are directly compared. This paper also uses different data analysis approaches and reporting structures, and therefore constitutes a distinct and independent publication.”

Please clarify whether this [conference proceeding or publication] was peer-reviewed and formally published. If this work was previously peer-reviewed and published, in the cover letter please provide the reason that this work does not constitute dual publication and should be included in the current manuscript.

- The following statement has been included in the cover letter: “The blood data presented in this manuscript have been previously reported in two separate peer-reviewed papers (attached as Paper 1 and Paper 2). The CRS data have also been reported in Paper 2, but in a different format. The current manuscript is the first to incorporate thoracic ultrasonography (TUS) data and to directly compare multiple methods within the same study. Additionally, it employs different data analysis approaches and reporting structures. Therefore, this manuscript constitutes a distinct and independent publication and does not represent dual publication.”

4) Please include captions for your Supporting Information files at the end of your manuscript, and update any in-text citations to match accordingly. Please see our Supporting Information guidelines for more information: <http://journals.plos.org/plosone/s/supporting-information>.

- We have included the following statement after the acknowledgements section: “**Supplementary information**

The raw dataset supporting the findings of this study is provided as Supplementary File S1.”

5) If the reviewer comments include a recommendation to cite specific previously published works, please review and evaluate these publications to determine whether they are relevant and should be cited. There is no requirement to cite these works unless the editor has indicated otherwise.

- N/A

*Dear Reviewer #1,*

*We sincerely thank you for the time and effort you have dedicated to reviewing our manuscript. We have carefully considered all your comments and revised the manuscript accordingly. Detailed, point-by-point responses to each comment are provided below. In particular, we have expanded the discussion on the grouping of TUS scores and further elaborated on the observed discordance between TUS and CRS to improve clarity and interpretability. We have attached a “clean” and “tracked changes” version of the document for your convenience.*

TUS and CRS comparisons with blood are made despite blood being sampled on *different days* than clinical assessments (T2, T4, T5). This can lead to inconsistencies and should be addressed with caution or sensitivity analysis.
Clarify this limitation in the methods more explicitly and consider whether statistical correction or subgroup analysis might mitigate this mismatch.
🡪 We have included additional text on the limitations of non-synchronous sampling in the discussion (L431).

Ensure past tense is used consistently in the methodology (e.g., "calves were scored" vs. "calves are scored")
🡪 We have had another read through and hope we have now changed the methods sufficiently to include only past tense statements. The present tense remains only where a figure is described.

Some values were excluded due to suspected lab errors (e.g., IgM, eosinophils). However, no mention is made of imputation or sensitivity analyses.
🡪 Thank you for your comment. We have adapted the exclusion criteria for outliers, and have now excluded values greater than *3SD above or below the mean (L208). The methods section and all resulting estimates have been updated.

Several sections (especially Discussion) contain overly long paragraphs. Breaking these into thematic blocks would improve clarity.
🡪 We have included some additional sub-sections in the discussion and hope this will clarify the readability.

L39: “...followed by a gradually decrease” → should be “a gradual decrease”
🡪 We think this sentence may have been misread, as it follows on the previous sentence which mentions ”CRS rapidly increased between T1 and T2 followed by a gradual decrease”. We have included an extra comma to clarify.

“...severe signs of respiratory disease” → could be simplified to “severe respiratory signs”
🡪 Thank you for your comment. This has been changed throughout the document.

"The CRS system was based on the Wisconsin calf health scorer..." → should be “based on the Wisconsin Calf Health Scoring Chart”
🡪 Now L161: We have updated this line.

Grouping TUS score 4 into score 3: This collapsing may affect results significantly given the small number of TUS 4 observations. A sensitivity analysis keeping TUS 4 separate, or pooling with discussion of clinical relevance, would improve rigor.
🡪 We have tried to analyse the data with TUS 3 and 4 kept separate, but due to the low number of observations of TUS 4, this did not provide additional relevance. We have included additional discussion on the clinical relevance in the limitations section of the discussion.

The kappa value (0.038) indicates **no meaningful agreement**, yet this is only discussed briefly. This result is central to the paper's conclusions
🡪 We have included additional discussion on this topic. (Line 423 - 435)

Include a clearer discussion of the CRS vs. TUS discordance and implications for field diagnosis.
🡪 Now L423 – 435: We have expanded the discussion on the TUS vs. CRS comparison.

*Dear Reviewer #2*

*We sincerely thank you for the time and effort you have dedicated to reviewing our manuscript. We have carefully considered all your comments and revised the manuscript accordingly. In particular, the statistical analyses have been updated to include a repeated effect for the blood measurements over time, as well as the inclusion of calf as a random effect in the analyses examining the associations between TUS, CRS, and blood variables. The corresponding text and tables have been amended to reflect these updates. Detailed, point-by-point responses are provided below. We have attached a “clean” and “tracked changes” version of the document for your convenience.*

L31 | "TUS scores (0: healthy to 3: severe consolidation)" – Incomplete scale; Table 2 shows scores up to 5. | Revise to: "TUS scores (0: healthy to 5: severe pneumonia)" and clarify in text that scores 4–5 were rare and collapsed into score 3 for analysis (L196–197). |
🡪 Now L31: We have revised this sentence to clarify values of TUS 4 and 5 were reclassified for the analysis.

L32 | "CRS (0: healthy to 3: severe clinical symptoms)" – Implies linear scale, but scoring is categorical. | Clarify: "CRS categories (0: healthy, 1: mild, 2: moderate, 3: severe)" to reflect ordinal nature.
🡪 Now L32 – L33: We have revised this sentence to your suggested wording.

L35 "3 or 7 days post-arrival (T3)" – Introduces variability without justification. | State: "T3 was defined as 3 or 7 days post-arrival due to logistical constraints; analysis adjusted for time."
🡪 We agree this is an issue, however we were not able to address it in the abstract due to the word limit. We have ensured it is mentioned clearly in the methods.

L41 "Total immunoglobulins and IgG were lower for TUS of 3" – IgG is part of total Ig; cannot be lower unless others decrease. | Recheck calculation: Total Ig = IgA + IgG + IgM. If IgG ↓ and total Ig ↓, clarify if IgA/IgM compensate.
🡪 Now L41: Thank you for your comment here. IgG was measured in g-L, whereas both IgA and IgM were measured in mg/L. Due to this different scale, IgA and IgM had minimal contributions to total IG, explaining why both IgG and Total IG decreased with higher TUS, regardless of changes in IgA and IgM.

L59 "Calves that are diseased pre-transport should not travel" – Assumes causality not tested. Temper: "Calves with clinical signs pre-transport may be at higher risk during transport."
🡪 Now L57 – L58: We have now reworded this sentence to:” Calves with clinical signs pre-transport may infect other calves and may be at higher risk of severe physiological decline and impaired welfare during transport [4]. “

L65 "CRS has low sensitivity (0.27–0.64)" – No citation for these values in transport context. | Cite source specifically for transport or pre-transport settings (e.g., Donlon et al. 2023, Ref 21).
🡪 Now L64: As far as I am aware, no study has tested the sensitivity of CRS in transport settings. Donlon et al. studied dairy heifers on farm. I have adjusted the text to reflect that these values represent on-farm assessments and not during transport.

L74 "TUS has sensitivities >0.85 and up to 0.94" – Cites [10,11], but [10] is subclinical, [11] lacks necropsy validation. Specify: "Sensitivities vary by population; in subclinical calves, Ollivett et al. [10] reported 0.85–0.94 vs. BAL, not necropsy."
🡪 Now L73 – L74: I think there might be some misunderstanding regarding these references. Ollivett (2015) assessed the sensitivities of both TUS and BALF, but compared them to subclinical cases of respiratory disease that were first assessed by clinical assessments and later verified by euthanasia and necropsies. They reported a sensitivity of 0.94 and specificity of 1.0 for TUS vs. Necropsies. Rabeling et al. (ref. 11) also euthanised case animals (diagnosed with more severe disease) and performed postmortem examinations of the lungs.

L89–94 Calves from farms and marts mixed without accounting for origin differences. Include "origin (farm vs. mart)" as a covariate in models or stratify analysis.
🡪 Now L88 – L94: This analysis was preliminary, with a very small sample size. When we attempted to include repeated measures and additional covariates, the models did not converge. Therefore, we treated the four time-points as independent observations, and we were mindful of this limitation in our discussion. We did include calf as random effect in the model to account for the calf effect as much as possible.
We did have information on source, cohort, age, weight, sex, and breed; however, including only a subset of these variables would have been inconsistent, so we opted for an “all or nothing” approach. For descriptive purposes, we presented changes over time, but these were not used in the statistical models. With a larger cohort, we would have included all covariates, but in this preliminary study, this was not feasible.

L95–98 Describes cohort differences (sex, age, breed) but no statistical adjustment. Adjust models for cohort (C1 vs C2) or include as random effect.
🡪 See previous comment.

L105 "Calves from livestock mart assessed on day of transport" – Timing differs from farm calves (L104: one day pre). Standardize or acknowledge bias in pre-transport assessment timing.
🡪 We have included an additional sentence in the limitations section of the discussion (L498 – 501) to mention the possible effect of sampling these calves on different days.

L122–124 All calves received antibiotics upon arrival – confounds disease progression. Acknowledge that metaphylaxis limits natural disease progression and affects TUS/CRS/blood interpretation.
🡪 Now L121 – 124: The antibiotic use was mentioned four times in various sections of the discussion. The further elaborate, I have included the following sentence in the limitations: ”Firstly, the batch antibiotic treatments in this study is likely to have limited disease progression, particularly limiting clinical signs of disease, though this is standard practice in veal systems, and therefore the presentation of the current data is still representative for calves transported long-distance to intensive veal systems. “

L130–133 TUS and CRS not performed on same day at T4/T5 (blood sampled 1–2 days apart). State: "Non-simultaneous sampling may reduce correlation; results interpreted with caution."
🡪 The antibiotic use was mentioned four times in various sections of the discussion. The further elaborate, I have included the following sentence in the limitations: ”Firstly, the batch antibiotic treatments in this study is likely to have limited disease progression, particularly limiting clinical signs of disease, though this is standard practice in veal systems, and therefore the presentation of the current data is still representative for calves transported long-distance to intensive veal systems. “

L134–136 No blood at T3, but TUS/CRS done – limits temporal comparison. | Justify gap and avoid claiming integrated assessment at T3.
🡪 Now L134 – 136: Thank you for your comment. In the text, I have clarified that T3 samples did not include blood samples due to logistical constraints. Even though this paper presents descriptive statistics in changes over time, the statistical analysis directly compared assessments methods, regardless of the time they were collected at. Therefore the lack of blood samples at T3 means this timepoint was omitted for TUS or CRS vs. Blood comparisons. There was no temporal assessments in the statistical analyses.

L147 "Depth of 11cm" – Not validated for calf thorax; may miss deep lesions. Cite justification or note potential for missed consolidations.
🡪 Ollivett (2018) reported a depth of 8 to 10 cm is appropriate for evaluating lungs in young cattle. The observer in this study was trained by J. Donlon who has used Ollivett’s system and is well practiced in using thoracic ultrasonography for determining lung disease in calves. The ultrasound machine used did not have a standard setting between 8 and 10 cm, thus 11cm was used to best identify lesions in the lungs of these unweaned animals.

L153 "6-tier TUS" – Inconsistent with Table 2 (6-point scale) and text (0–3 used). Correct: "A 6-point TUS scale was used, but scores 4–5 were combined with 3 due to low frequency."
🡪 Now L152 – 157: We have reworded this sentence to clarify the original observations used a 6-point scale.

L161–163 | Rectal temperature omitted at T1 and imputed at T4 – introduces bias. Exclude temperature from T1 CRS or use alternative scoring; do not impute without validation.
🡪 Now L162 – 163: Temperatures were never measured at T1, and therefore were not present at the CRS calculation for this timepoint. They were not imputed. For T4, as temperatures were recorded within 24h of CRS assessments, and due to the gradual disease pattern, we believed it was most reliable to still include these variables. This was however thoroughly included as a limitation in the discussion.

L167–168 CRS categories based on sum 0–1=0, 2–3=1, etc. – unequal binning. Justify binning or cite Wisconsin system for this grouping.
🡪 Now L168 – 171: Thank you for your comment. We have included the reasoning for the binning in this sentence, it now reads: ”To equally compare CRS and TUS, an equal number of categories was favoured, therefore, total CRS scores were confined to four categories, a total score of 0 or 1 = CRS of 0, a total score of 2 or 3 = CRS of 1, a total score of 4 or 5 = CRS of 2 and a total score ≥ 6 = CRS of 3. “

L177 "26ml blood" – excessive volume for young calves (~28d). Confirm volume is safe (<10% total blood volume); typical calf blood volume ~80 mL/kg. 26 mL from 55kg calf = ~0.5% – acceptable, but state safety.
🡪 Now L179: I have included a sentence to state the % of total blood volume collected using a conservative 75ml/kg total blood volume, it now reads: ”For every blood sample, 26ml was taken by jugular venepuncture using a 20G needle by experienced personnel. This corresponds to 0.63% of blood volume for a 55 kg calf and 0.81% for the lightest calf (43kg), assuming total blood volume is 75 mL/kg for a calf between 16 and 30 days [20], both below the 1% safety threshold for sampling of total blood volume within 24h [21]. “

L182–183 Two hematology analyzers used (Advia 2120, XT-1800i) – may introduce batch effects. Perform cross-calibration or include analyzer as covariate in models.
🡪 Now L186 – 188: Assay performance was analysed at both locations, we have now included the following text: ”Assay performance was ensured through the inclusion of quality control samples, for Teagasc Grange samples %CV was calculated in low, normal, and high quality control samples for HGB (low: 1.31%, normal: 0.89% and high: 1.73%) and neutrophil count (low: 4.75%, normal: 2.33%, and high: 3.21%). For Rimondia samples, only normal quality control samples were included, which yielded a %CV of 0.78% for HGB and 3.20% for neutrophil count. “

L187 Hb units converted (g/dL to mmol/L) – conversion factor not cited. Cite: "Hb converted using factor 0.6205 (mmol/L per g/dL) [Ref]."
🡪 Now L196 – 197: We have been unable to find referenced directly citing conversion ratios, and therefore looked up several conversion calculator websites, as all mentioned the same conversion ratio (0.6206), we cited one of these websites as a reference. We also noted a numerical error in the text (0.6205 = 0.6206) which we corrected.

L195–196 One calf removed due to missing TUS – violates intention-to-treat. Report as missing data and use imputation or sensitivity analysis.
🡪 Now L204 – 205: We understand your point here, however, missing this datapoint in this one calf meant that descriptive statistics and the analysis in changes in TUS over time were incomplete and inaccurately calculated unless omitting its data. We believe it was most appropriate to remove this calf’s data from the dataset.

L196–197 TUS 4 reclassified as 3 – alters disease severity. Report both ways: primary analysis with TUS 0–3, secondary with TUS 4 separate.
🡪 We understand your comment here, but have thus far opted to keep the reclassification of TUS = 4 as TUS = 3 instead. As a TUS of 4 was only observed on four occasions, the power of this category was too low to show reliable estimates, which would not add any value to the results of this study. Many researchers using TUS actually use TUS consolidation as a single category, rather than classifying based on the location of the lesion (i.e. Jourquin et al. 2024, Buczinski et al. 2015 and 2016, and Berman et al, 2020 and 2021), which further supports our decision to class any cases of consolidations equally for the statistical analysis. We have however changed the text to use a category of TUS ”≥” 3 in the text and tables. And we have further defended the pooling of TUS values in the discussion.

L198–200 Outliers removed without statistical justification (e.g., >3 SD). Define outlier criteria (e.g., >3 IQR) and report number excluded.
🡪 Now L208 – 209: Using IQR removed more values than necessary, we instead opted to remove values more than 3*SD above the mean for these variables and have updated the text and results accordingly.

L207–212 "Minimal model" not defined; no random effects for repeated measures. Use mixed models with calf ID as random effect to account for repeated measures.
🡪 Now L215: Thank you for your comment. We have now included repeated measures with calf as the subject and have updated all text below accordingly.

L212 Residuals tested for normality – but no mention of transformation validation. Show Q-Q plots or Shapiro-Wilk results in supplement
🡪 Now L220: We believe attaching the Q-Q plots in the supplementary data is excessive, however, we have included the plots at the end in the ”reviewers responses” document for your review.

L214–220 Different transformations per variable – but no consistency in back-transformation. Ensure all SEs and CIs are correctly back-transformed (L224–229 are correct; verify in tables).
🡪We believe the section on back transformations may have been missed. It is included in the last paragraph of the statistical analysis section in the methods chapter (L231 – 238).

L222 Weighted kappa used – appropriate, but no confidence interval reported. Add 95% CI for kappa in text or figure.
🡪 We have included the 95% CI in the text (L360).

L241 "No calves died" – but mortality is relevant to BRD severity. Discuss survival bias: severe cases may have been treated early.
🡪 Now L249: We agree calves were treated early, which likely reduced severe respiratory disease. Some text in the limitations section (discussion) was added to discuss this issue. We have also added the following sentence to further explain the absence of mortalities in the study calves. ”Early treatment is also likely why no calves died and may have reduced the occurrence of severe respiratory disease in the study calves. ”

L243 "67% had TUS ≥2" – implies disease, but no validation against gold standard. Use: "67% had ultrasound findings suggestive of lung pathology."
🡪 Now L251 – 252: We have changed the text to include your suggestion.

L244–248 Location of lesions described, but not analyzed statistically. Report if right cranial lobe predominance is significant (e.g., chi-square).
🡪 Now L252 – 257: Thank you for your comment. We totally agree that the location of lesions is important, but this is one of the main objectives for our next paper, which is why it is not discussed in detail in this manuscript.

L257 "Chi-square; p=0.03" – Chi-square for transition frequencies? Inappropriate. Use McNemar’s test or generalized estimating equations (GEE) for paired ordinal data.
🡪 Now L266: We have worked on these statistics once again as we agree the chi-square analysis was not appropriate. However, the McNemar’s test requires binary data, and our data has three ordinal categories. The GEE model would be more appropriate, but requires individual datapoints while our objective at this stage was to show rough changes in TUS progression between timepoints rather than an in-depth analysis. We believe sticking solely to descriptive statistics in this section is more appropriate and have changed the text accordingly.

L289 "Chi-square; p<0.01" – same error as L257. Replace with GEE or marginal model for longitudinal CRS changes.
🡪 Now L297: We also changed the text here to reflect solely descriptive statistics.

L307 "9 out of 14 variables changed" – cherry-picking; no adjustment for multiple testing. Apply FDR correction (e.g., Benjamini-Hochberg) and report q-values.
🡪 Now L314: We believe our sentence here may have been misunderstood. All fourteen variables were independently tested compared to the timepoints they were measured at. Our inclusion of the "9 out of 14 variables changed" is solely to help the reader understand more easily that a large number of variables changed during or after transport. This sentence leads into the next sentence where differences between individual variables and timepoints are further discussed. We hope you agree that this does not require additional analysis.

L308 "Neutrophil/lymphocyte ratio decreased between T4 and T5" – contradicts Table 4 (T3 missing). Correct: "N/L ratio decreased from T1 to T5" or clarify trend.
🡪 Now L316: This has been reworded to ”neutrophil count decreased after T4, T3 was not mentioned. As a result of statistical changes, this table and text has also been updated.

For the following comments:

- L323 "Lymphocyte count was lower for TUS of 3 than any lower TUS" – but TUS 0 vs 1 not different? Specify: "significantly lower than TUS 0, 1, and 2".
- L324 "N/L ratio higher for TUS 3 vs 0 or 2" – but p=0.03 and 0.01 – significant, but not vs 1? Clarify: "not significantly different from TUS 1".
- L325 "SAA higher for TUS 2 or 3 than TUS 1" – but Table 5 shows TUS 1=49.9, TUS 2=65.4, TUS 3=73.3. Correct: "higher for TUS ≥2 than TUS 0 or 1".
- L326 "Total Ig lower for TUS 3 than TUS 2" – but TUS 1=14.2, TUS 2=16.4 – TUS 1 not different? State: "significantly lower than TUS 2", not "any lower TUS".
- L322 "Haemoglobin did not show differences despite significant p=0.04" – contradiction. Recheck: p=0.04 is significant; either remove "did not show" or correct p-value.
- L344 "Eosinophil count did not show differences despite p=0.05" – p=0.05 is significant. Correct: "marginally significant" or adjust alpha.

🡪 These sections on TUS and CRS versus blood variables have been revised following updates to the statistical model, particularly the inclusion of calf as a random effect. We have further clarified references to non-significant differences in the least-squares means comparisons, which previously caused some confusion, and have included p-values to indicate where significant differences were observed.

L344 "Eosinophil count did not show differences despite p=0.05" – p=0.05 is significant. Correct: "marginally significant" or adjust alpha.
🡪 Now L347 – 348: Thank you for your comment. I believe some confusion may have occurred here. Eosinophil count showed a significant p-value (0.0453), but least-square means comparisons of individual CRS scores, using Tukey-adjusted p-values, were not significant. I have updated the sentence here to clarify.

L355 "Severe TUS never correlated to severe CRS" – but n may be too small. Add: "though low frequency of severe cases limits inference."
🡪 We agree more discussion regarding this low agreement had to be included, and we have included more on this topic in the discussion. We did not feel it was appropriate to include in the results section.

L377 "almost 50% had some inflammation" – but TUS 1 may not be pathological. Use: "ultrasound abnormalities", not "inflammation", without histology.
🡪 Now L383-384: We have revised this sentence to ”The fact that pre-transport ultrasound scanning revealed abnormalities in the lungs of almost 50 % of calves.. “.

L380 "prevalence of severe lesions 4.8%" – based on observations, not calves. Report per calf: "4.8% of calves had at least one TUS=3 or 4 observation."
🡪 Now L386: We have reworded this sentence, and updated the percentage to 4.7%, 4.8% was unfortunately written in error. It was unclear that this sentence was focused on percentages pre-transport, which has now been clarified.

L382 "72% of study calves displayed lung lesions" – at what time? T3? Specify: "at T3, 72% of calves had TUS ≥1."
🡪 Now L389: We have changed the sentence to clarify this point. It now reads ”Overall, the deviation from health in transported calves peaked at approximately 7 days after transport, as indicated by the fact that 72 % of study calves displayed had a TUS ≥ 1 at T3, despite antibiotic interventions during this period. “

L388 "consolidation to fully recover" – assumes reversibility without histology. Say: "ultrasound lesions resolved", not "fully recover".
🡪 Now L394: We have changed the wording here to ”a potential for ultrasound lesions to resolve over time…”

L394 "failure of ultrasound technique" – contradicts claim of high sensitivity. Replace: "inter-observer variability or transient lesions may explain changes."
🡪 Now L398 – 401: We have changed this sentence and included transient lesions as a potential explanation. Inter-observer reliability was not applicable as a single observer performed all observations.

L400 "rectal temperatures omitted... due to practical concerns" – weakens CRS validity. Acknowledge CRS is incomplete pre-transport and may underestimate disease.
🡪 Thank you for your comment. We have added additional information after this text, it now reads: ”It must be noted that CRS calculations pre-transport did not include rectal temperatures. These were omitted from observations due to practical concerns; however, even when omitting rectal temperatures from CRS calculations at all time points, the general pattern of change remained similar, with a rapid increase in CRS values between T1 and T2 and a gradual decrease thereafter. **Regardless, the incomplete calculation of CRS pre-transport may have underestimated disease.** “

L416–418 "174 observations of healthy CRS, 104 not healthy by TUS" – but TUS may detect subclinical disease. Reframe: "TUS detected abnormalities in 60% of clinically healthy calves."
🡪 Now L425 – 435: We have changed this sentence and incorporated your suggestion.

L430 "haemoglobin usually increases" – incorrect; Hb often decreases in BRD due to anemia of inflammation. Correct: "In contrast to some reports, haemoglobin decreased, possibly due to acute phase response."
🡪 Now L443 – 444: We have been unable to find clear publications reporting a decrease in haemoglobin values in response to BRD in unweaned dairy calves. We have toned down the statement here and also included a reference reporting a decrease in haemoglobin in (non-specified origin) calves.

L439 "Low total Ig and IgG... more at risk" – cites [16], but [16] is about arrival Ig predicting disease. Accurate: "As shown by Pardon et al. [16], low Ig at arrival predicts later BRD."
🡪 Now L451: We have incorporated your suggestion and changed the sentence accordingly.

L463 "TUS requires up to five minutes" – but no data in manuscript supports this. Add: "Based on observer logs, average scan time was X minutes."
🡪 Now L473: We have slightly changed the statement here, and have included a reference instead of our own observer logs.

L465 "scanning right cranial lobe captures 61% of severe scores" – useful, but not validated. Recommend: "may be sufficient for screening, pending validation."
🡪 Now L478 – 479: We have reworded this sentence to clarify validation is still needed.

L476–478 Blood and TUS/CRS not simultaneous – major limitation for correlation. State: "non-synchronous sampling likely attenuated correlations between blood and clinical scores."
🡪 Now L494 – 498: We have incorporated your suggestion into the sentence.

L481–482 "suboptimal situations" – but no description of how this affected blinding or consistency. Describe environmental constraints and their potential impact on scoring reliability.
🡪 Now L501: We have clarified that the suboptimal conditions referred to the next sentence (the farm environment, in which calves were housed individually, made TUS observations complicated due to limited space for the observer in the pen next to the calf”. As the data in this trial was collected observationally, blinding was not applicable. Post-arrival, all calves were housed in similar pens and similar farm environments, which assisted in optimizing scoring technique consistency.

L484 "observers wear respiratory masks" – safety note, not research finding. Move to methods or supplementary.
🡪 We have removed this sentence from the discussion. We do not feel it suits in the methods, and prefer to exclude the statement.

L485 "study increased stress" – confounder not measured or adjusted for. Measure cortisol or behavior, or acknowledge as limitation.
🡪 L507: This sentence is mentioned in the limitations section of the discussion. As it is mentioned as a limitation we have currently not made any additional changes.

L487–497 Conclusion: "TUS should be compared to other methods" – already done. Reframe: "TUS showed better alignment with immune markers than CRS, supporting its use in high-risk settings."
🡪 Now L516 – 517: Thank you for your comment, we have included your suggestion and amended the sentence accordingly.

**Q-Q plots for Blood versus Timepoints.**

**Q-Q plots for TUS versus Blood.**

**Q-Q plots for CRS versus Blood.**
